# Developing Healthy Food Preferences in Preschool Children Through Taste Exposure, Sensory Learning, and Nutrition Education

**DOI:** 10.1007/s13679-018-0297-8

**Published:** 2018-02-14

**Authors:** Chandani Nekitsing, Marion M. Hetherington, Pam Blundell-Birtill

**Affiliations:** School of Psychology, University of Leeds, Leeds, LS2 9JT UK

**Keywords:** Healthy eating intervention, Experiential learning, Taste exposure, Education intervention, Vegetable intake, Fussy eating

## Abstract

**Purpose of Review:**

The present review was undertaken in order to summarize and evaluate recent research investigating taste exposure, sensory learning, and nutrition education interventions for promoting vegetable intake in preschool children.

**Recent Findings:**

Overall, taste exposure interventions yielded the best outcomes for increasing vegetable intake in early childhood. Evidence from sensory learning strategies such as visual exposure and experiential learning also show some success. While nutrition education remains the most common approach used in preschool settings, additional elements are needed to strengthen the educational program for increasing vegetable intake. There is a substantial gap in the evidence base to promote vegetable intake in food fussy children.

**Summary:**

The present review reveals the relative importance of different intervention strategies for promoting vegetable intake. To strengthen intervention effects for improving vegetable intake in preschool children, future research could consider integrating taste exposure and sensory learning strategies with nutrition education within the preschool curriculum.

## Introduction

### Developing Food Preferences Early Is Important

Early childhood is a period of rapid growth and an important phase for developing eating habits because the dietary behaviors acquired during the early years of life can extend to adulthood [1–3]. Children learn about their food likes and dislikes by direct contact with foods, such as through tasting, feeling, seeing, and smelling, and also by observing their food environment, for example, the eating behaviors of others [4–6]. The significant rise in children with obesity by the time they start school is of public health concern [7]. In the UK, Health Survey for England (HSE) 2014, has reported that 17% of children aged 2–15 years are currently with obesity and a further 14% with overweight [8]. Similar figures are also reported in the USA; the 2013–2014 National Health and Nutrition Examination Survey indicated 17% children and adolescents aged 2–19 years with obesity, in addition to 16% with overweight [9].

Preventing excess adiposity includes increasing physical activity and matching dietary intake to energy requirements. Eating a well-balanced diet which is high in intake of fruits and vegetables (minimum 200 g/day for children and 400 g/day for adults) is thought to reduce the risk of obesity but also prevent various non-communicable diseases such as type 2 diabetes, cancers, and cardiovascular diseases [10, 11]. Vegetables in particular are beneficial because they are low in naturally occurring sugars compared to fruits [12]. Studies with a largely middle-aged population indicated that eating vegetables confers a protective effect with each daily portion consumed reducing the probability of death by 16%, compared to 4% for fruits [13]. Most children in the USA and UK eat far below the recommended amount of fruits and vegetables [14, 15] and increasing intake of vegetables remains challenging due to their bitter taste, texture, and low energy density. Also, eating behavior traits such as food fussiness and food neophobia can affect intake. Despite the various contributing factors for children’s low consumption and dislike of many vegetables, the early years is a key period in development to encourage acceptance through appropriate behavioral techniques such as repeated taste exposure. The aim of the present review was to assess the relative importance of taste exposure, sensory learning, and nutrition education interventions to promote vegetable intake in preschool children; and to consider the ways in which vegetable intake and liking can be encouraged in fussy eaters.

### The Role of Food Fussiness and Food Neophobia

Food fussiness is defined as selective food intake involving rejection of foods which are familiar as well as those which are unfamiliar, whereas food neophobia is specific to avoidance of new foods [16]. Food fussiness is typically measured using the Food Fussiness subscale of the Child Eating Behaviour Questionnaire [17] and food neophobia is often assessed using the Child Food Neophobia Scale [18]. These psychometric tools are reported to be reliable and valid; however, they tend to overlap in constructs which can be problematic when using them together. Both eating behaviors can have an adverse effect on healthy eating, since by selecting only preferred familiar foods, children may limit the quality and variety of their diet, especially by not eating sufficient vegetables [16, 19]. Food fussiness and food neophobia are highly correlated (with *r* values around 0.7) and they both share common etiology in early childhood, which can be explained by heritability and shared environment factors, such as the home environment [20]. Food avoidance behaviors have been associated with low intake of fruits and vegetables [19, 21], and a recent study by Fildes et al. [22••] found that genes common to food fussiness also influence the intake of fruits and vegetables. It is understood that rejection of certain foods such as bitter vegetables and development of food avoidance behaviors are a consequence of natural evolutionary processes in which children’s instinctive behavior is to avoid potentially harmful substances [23, 24]. Nonetheless, it is important for children to include bitter, green leafy vegetables in the diet to promote health.

Food fussiness and food neophobia are known to peak between the ages 2 and 5 years; however, children in this age group are also open to acquiring new food preferences. Therefore, encouraging children using taste exposure and experiential learning strategies in a positive and supportive environment can promote familiarization and liking of unfamiliar foods, and over time, this may reduce their food avoidance behaviors [2, 25].

### Repeated Taste Exposure Interventions

Studies of complementary feeding identify early flavor exposure to vegetables as an important strategy during the weaning stage (around 6 months) to encourage acceptance [26, 27]. However, for some children, the taste and smell of some foods leads to a negative evaluation and hence to food refusal. Due to their bitter taste, this can be a particular problem when children eat vegetables. [28]. The so-called repeated taste exposure strategy^1^ in which children are offered the same foods frequently is reported to be the most effective way of promoting intake of unfamiliar vegetables in children [29••, 30–32]. The strategy works by a process of familiarization [33] and learned safety [34]. According to the mere exposure theory, a single exposure is sufficient to produce a positive attitude towards a stimulus; thus, repeated taste exposure interventions promote positive acceptance over time [33, 35]. Studies have demonstrated that children increase their intake of vegetables after five exposures; however, on average, children will generally require between eight to ten exposures at a regular interval, e.g., once a week [31, 36–39]. The required number of exposures is often not achieved by the caregiver because they may interpret their child’s facial expression as genuine dislike and therefore are unwilling to continue offering the same food [40–42].

A number of studies have been carried out using repeated taste exposures with other strategies such as non-food rewards (such as sticker or praise) [38, 43•, 44], modelling (learning by observing others, e.g., parents or peers) [38, 44], flavor-flavor learning (pairing with liked food or flavor, e.g., apple sauce or salt) [32, 45, 46], and flavor-nutrient learning (added energy, e.g., oil) [36, 45]. A study of reward and taste exposure by Fildes et al. [43•] found that parental administration of a single small piece of the disliked vegetables daily with a sticker was sufficient to increase the intake of a target vegetable in a home setting. Combining taste exposure with strategies such as rewards and modelling have long-lasting effects, up to 6 months post-intervention [44, 47]. However, Horne et al. [44] found that once liking for the target foods was established during snack time, the effects generalized to lunchtime in complete absence of reward. Most studies incorporating associative learning have found that mere exposure to the target vegetable alone is sufficient to increase the intake of the target vegetables, and adding flavors or nutrients provide no additional advantage [36, 45, 46, 48]. The exception to this observation was reported in a study showing that exposure alone was effective for a non-bitter familiar vegetable (cauliflower). However, adding cream cheese was more effective for increasing liking of an unfamiliar bitter vegetable (Brussels sprouts) [49].

While the taste exposure strategy has a robust outcome for promoting vegetable intake in children, it should be noted that when a child tastes a food, taste is not the only sensory feature that they are exposed to. They also engage with the food using other non-taste sensory modalities such as hearing (the name of the food or sound it makes while chewing), sight (seeing the food), touch (feeling the texture in hands/ mouth), and smell of the food [24].

### Non-taste Sensory Learning Interventions: Sound, Sight, Touch, and Smell

During complementary feeding, parents are advised to incorporate finger foods at mealtimes in order to encourage their children to experience new food textures. The hands-on learning which occurs during this time may facilitate acceptance of new and varied foods during this period. However, as children make the transition to the family diet, they move away from ‘hands on’ sensory exploration towards eating with spoon or fork and may be discouraged from ‘playing with their food’ at meal times.

Contemporary research with preschool children suggests that interventions could include non-taste sensory elements to familiarize children with fruits and vegetables [50••, 51, 52•]. Although their outcomes are not as favorable as taste exposure interventions, methods including listening, seeing, touching, and smelling may be very useful for young children, especially picky eaters as selective eating in preschool children has been associated with hypersensitivity to some of these senses [24, 52•, 53]. For example, food neophobic children and fussy eaters often reject vegetables based on their visual appearance or texture [16, 35, 52•, 54, 55]. Hence, interventions incorporating visual exposure including picture books and tactile-play activities may particularly benefit these children more than taste exposure alone.

#### Visual Exposure and Narrative (Sound and Sight)

Listening to stories and looking at pictures are activities which are regularly enjoyed by preschool children. Storybooks are generally engaging and interaction with the parent or caregiver during story time provides an opportunity for children to acquire new knowledge [56]. The illustrations in storybooks help children to better recall stories and being repeatedly exposed to pictures of foods increases children’s visual familiarity with the foods [57, 58•]. Research on visual exposure using picture books has had mixed results in terms of their effectiveness in increasing acceptance of vegetables [58•, 59–62]. For example, Heath et al. [58•] reported that toddlers aged 19–26 months who were exposed to a storybook every day for 2 weeks consumed more of an unfamiliar vegetable that they had been visually exposed to compare to an unexposed control vegetable. The picture book exposure not only increased the intake of the unfamiliar vegetable, but it also reduced the level of encouragement needed for the children to taste the target foods. In comparison, de Droog et al. [62] reported a positive effect of exposure to a picture book (which included a narrative embedded with a health message) on intake of a familiar vegetable. They further added that picture books are particularly effective when the children are actively involved (e.g., when asked questions about the story). Children aged 4–6 years increased their intake of carrots more after being exposed to the vegetable in a picture book. The study also found that eating more carrots displaced consumption of cheese (high-calorie food). In contrast, a study with 4–8-year-old children found that engaging children with real vegetables produced a stronger effect on willingness to taste these vegetables (fresh soya bean) than visual exposure using photographs [51]. However, it should be noted that this study did not include a control group and perhaps visual exposure could increase willingness to taste relative to no exposure at all.

The main advantage of using this strategy is that it can be implemented outside of the meal context. This may alleviate the stress associated with tasting the vegetables for some children [24]. Other advantages of visual exposure using storybooks include ease of administration, social interaction between carers and children, engaging in a learning exercise to promote knowledge and awareness, and setting social norms. The many beneficial effects of visual exposure studies has given rise to research on multisensory learning for improving vegetable intake in children [51, 52•, 54, 63–65].

#### Role of Olfaction (Smell) in Food Intake

Olfaction plays an important role in sensing of foods; the smell of the food contributes to overall flavor experience and can influence the desire to consume a particular food [66]. In adults who were restrained eaters, exposure to the odor of pizza increased intake, liking, and desire to eat that food [67]. In comparison, a study by Ramaekers et al. [68] with healthy-weight women reported that sniffing banana odor increased appetite for banana, although it did not influence the overall food intake. There are some inconsistences in findings reported from olfaction studies. However, the influence of odor on less palatable/less pungent foods such as raw vegetables is understudied and warrants further investigation. It should be noted that the outcome of olfactory activities may also depend on individual’s awareness and ability to smell, both of which can vary by age [69, 70]. Although there are no studies implementing olfactory experience alone for vegetable intake in children, there is emerging evidence for its use in multisensory interventions [24].

#### Experiential Learning Intervention (Sound, Sight, Touch, and Smell)

**Fig. 1:**
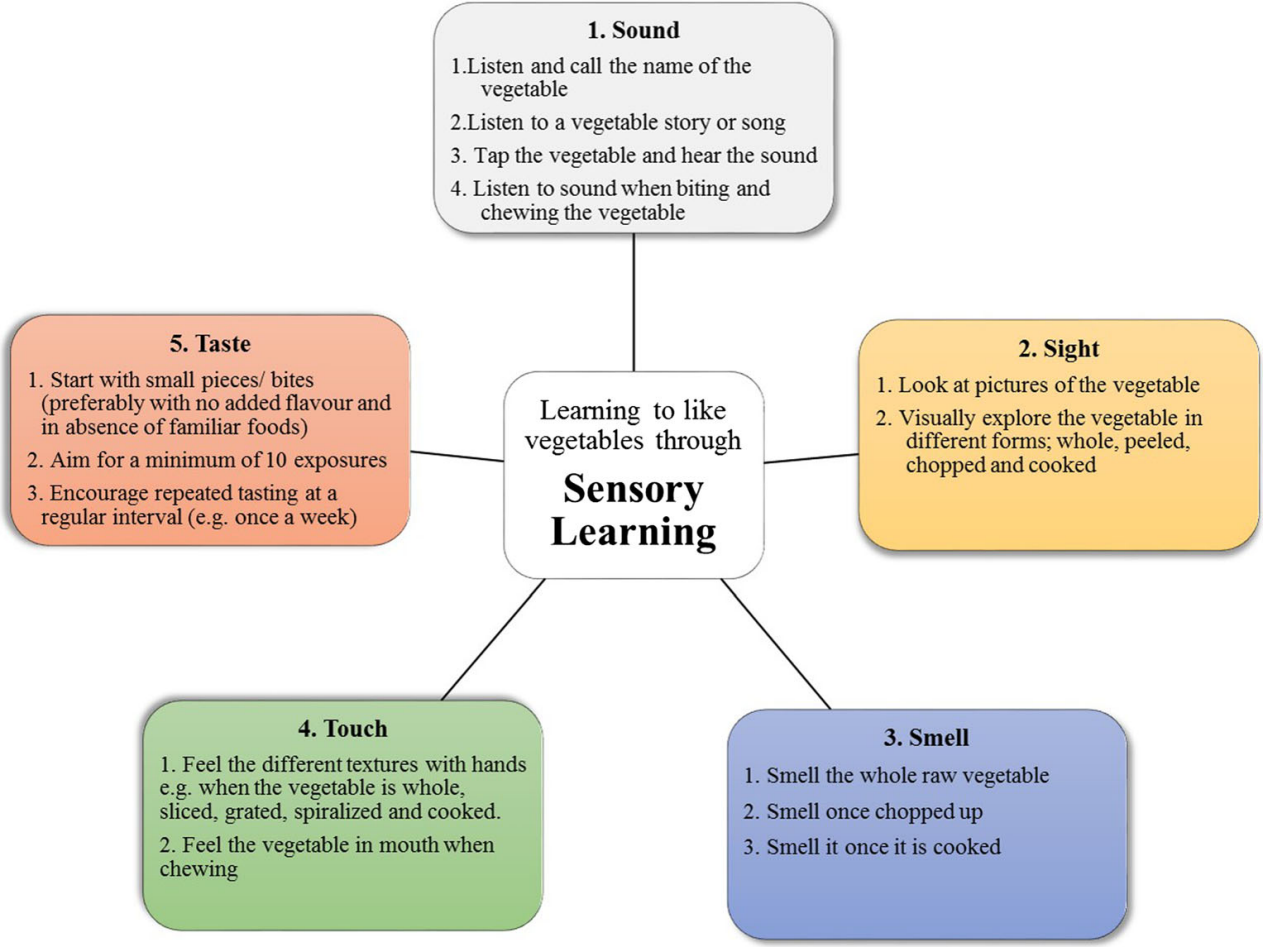
Basic sensory learning techniques that parents or preschool carers can use to encourage young children to become familiar with foods. Ideas adapted from work by Dazeley and Houston-Price [24]

Sensory activities with food involving listening, seeing, smelling, touching, and tasting can be encouraged from a very young age (see Fig. 1 for simple techniques which can be used when introducing new foods to young children (ideas adapted from Dazeley et al. [24])). These learning strategies can be incorporated during the usual mealtimes or outside of the mealtime context, e.g., during cooking, gardening, and nutrition education sessions. For example, ‘Taste for Life’ is an intervention based on sensory learning which was developed to support nurseries in encouraging young children to eat healthily [71]. The program emphasizes having fun with fruits and vegetables outside of meal contexts, using different senses such as touch (handle, see, and smell), taste, and sound (song and rhyme time) [50••]. However, it is not yet clear if this leads to changes in intake. Therefore, more research is needed to evaluate the effectiveness of existing programs, specifically how successful and feasible they are for improving vegetable intake.

Where studies have been conducted on multisensory learning (using sight, sound touch, smell, and excluding taste) as a way of familiarizing children to new foods, evidence suggests these are successful for increasing fruit and vegetable consumption. For example, a study in children aged 12–36 months found that familiarizing children to unfamiliar fruits and vegetables by looking, listening, feeling, and smelling them during playtime for 4 weeks increased their willingness to touch and taste these foods during lunch time [24]. Similarly, Coulthard and Sealy [52•] found that a single session of sensory play with fruit and vegetables was enough to encourage children to try more of the study foods than those who only observed the fruit and vegetable sensory play session or received non-food sensory learning, demonstrating the importance of children actively touching and feeling the foods. However, this study only examined the effects on familiar vegetables (i.e., carrots, cucumber, and tomatoes [see, 72]), which may not generalize to unfamiliar vegetables.

Interacting with the sensory properties of food during tactile-play may particularly benefit children who are food neophobic, going through a period of fussy eating or who are simply unwilling to taste new/disliked foods [50••, 52•, 54, 65]. However, these suggestions are only based on the correlations observed between food avoidance behaviors, sensory learning, and vegetable intake and therefore warrant further investigation. In particular, it should be noted that children who are picky/food fussy or neophobic may also be more sensitive to touch and therefore may dislike these types of activities.

### Nutrition Education Interventions

Parents play a central role in their child’s eating habits; however, as many young children regularly attend childcare, preschool settings are ideal for encouraging children to eat healthily [73•, 74]. In order to prevent obesity, public health interventions encourage preschool settings to promote healthy eating, in particular, to increase the consumption of fruits and vegetables. As a result of this, most day-care settings are required to integrate some nutrition education within the preschool curriculum [75]. Nutrition education programs in the early years’ settings teach day-care staff, parents, and/or children about the nutritional value of food [76–78]. Interventions which involved parents generally involved giving them nutritional information. For example, Sharma et al. [79] sent a tip-sheet about modifying home nutrition, whereas Sirikulchayanonta et al. [80] provided a letter with guidance to motivate and encourage children to eat variety and quantity of fruits and vegetables, and Tabak et al. [81] gave parents the option to choose one of four newsletters from the following topics: vegetable availability, picky eating, modelling, or family meals. In education programs where children are involved, the sessions are usually interactive and engaging as they incorporate fun activities such as educational stories, drawing, games, gardening, cooking, and tasting [79, 82, 83].

Nutrition education programs vary in duration from a few weeks to several months, and they usually aim to increase consumption of familiar fruits and vegetables. While they are often successful in increasing vegetable intake in children, the effect sizes are smaller than other interventions such as sensory learning, reward, or taste exposure [29••, 84, 85••]. One reason for the weaker outcomes may be the over-reliance on self-report (food frequency questionnaires) or assessing proxy measures of intake, such as liking, knowledge, and willingness to taste. More research with accurate intake data is needed, e.g., weighed intake in grams. Another reason why nutrition education may not be as effective as interventions involving hands-on experience is because children gain indirect experience through the curriculum rather than direct exposure to foods by smelling, feeling, and tasting. Therefore, incorporating sensory activities including taste to nutrition programs may provide an opportunity for children to improve both their knowledge and intake of vegetables.

### General Discussion and Future Research

Evidence suggests that most published interventions that aim to improve vegetable intake are successful to some extent. However, they all have some limitations which should be considered when developing future interventions [85••]. Education is crucial for building knowledge about eating a variety of vegetables; however, simply learning about why and what we should eat does not bridge the gap between awareness and actual consumption. The effects of repeated taste exposure using a single vegetable are robust and durable. However, there is no evidence for its effect beyond the single target vegetable [86••]. Taste may be the most crucial sensory element when encouraging children to eat vegetables, but other sensory properties may be important as well. The emerging evidence from non-taste sensory learning is promising but the long-term impact of these strategies remains unknown [24]. Both taste exposure and non-taste sensory experience are more effective for increasing intake of unfamiliar vegetables compared to familiar vegetables. This may be a result of ceiling effects—it is difficult to increase the liking of vegetables that are already familiar and liked. Some generalization effects have been observed with taste exposure to a variety of vegetables [44] and this may also be relevant in future sensory interventions. More research is needed to understand if taste and experiential learning using a variety of vegetables can generalize to other vegetables and other settings (e.g., from preschool to home). Also, in order to sustain the change in intake of vegetables in the longer term, research could be conducted to investigate lasting effects of interventions.

It is often difficult to engage parents in nutrition education programs which are based in preschools. Therefore, there is a need for parental and preschool staff process evaluation which could help to build connections between providers and parents to improve children’s nutrition [87]). A single strategy is unlikely to work for every child; therefore, combining strategies discussed earlier rather than using a single component intervention may benefit greater numbers of children, including those who are going through a period of picky eating. In particular, more research is needed to understand which strategies work for food fussy children.

## Conclusions

Insufficient intake of vegetables in children remains an area of concern for parents and public health agencies. In order to improve children’s nutrition, it is important that they eat the recommended quantity of vegetables. Repeated taste exposure strategies are the best evidenced for increasing intake of unfamiliar vegetables. However, there may be a role of experiential learning and nutrition education to expand the child’s knowledge, awareness, and willingness to taste and also eat vegetables. Therefore, future research could identify the most effective elements of these strategies and integrate them to produce stronger and more durable outcomes for increasing vegetable intake in the early years.

## Footnotes

1 It is understood that taste exposures involves more than just taste since children see, smell, and taste the food; however, many interventions refer to taste exposure rather than visual and flavor exposure.
